# Oyster-Derived Tyr-Ala (YA) Peptide Prevents Lipopolysaccharide/D-Galactosamine-Induced Acute Liver Failure by Suppressing Inflammatory, Apoptotic, Ferroptotic, and Pyroptotic Signals

**DOI:** 10.3390/md19110614

**Published:** 2021-10-28

**Authors:** Adrian S. Siregar, Marie Merci Nyiramana, Eun-Jin Kim, Soo Buem Cho, Min Seok Woo, Dong Kun Lee, Seong-Geun Hong, Jaehee Han, Sang Soo Kang, Deok Ryong Kim, Yeung Joon Choi, Dawon Kang

**Affiliations:** 1 Department of Physiology and Institute of Health Sciences, College of Medicine, Gyeongsang National University, Jinju 52727, Korea; adriansiregar46@gmail.com (A.S.S.); mariemerci1994@naver.com (M.M.N.); eunjin1981@hanmail.net (E.-J.K.); whitewms@naver.com (M.S.W.); dklee@gnu.ac.kr (D.K.L.); hong149@gnu.ac.kr (S.-G.H.); jheehan@gnu.ac.kr (J.H.); 2 Department of Convergence Medical Science, Gyeongsang National University, Jinju 52727, Korea; kangss@gnu.ac.kr (S.S.K.); drkim@gnu.ac.kr (D.R.K.); 3 Department of Radiology, Ewha Womans University Seoul Hospital, Seoul 07804, Korea; kingnose80@gmail.com; 4 Department of Anatomy and Institute of Health Sciences, College of Medicine, Gyeongsang National University, Jinju 52727, Korea; 5 Department of Biochemistry, College of Medicine, Gyeongsang National University, Jinju 52727, Korea; 6 Ocean-Pep, Jinju Bioindustry Foundation, Jinju 52839, Korea; yjchoi@gnu.ac.kr

**Keywords:** acute liver injury, apoptosis, ferroptosis, inflammation, oyster, peptide, pyroptosis

## Abstract

Models created by the intraperitoneal injection of lipopolysaccharide (LPS) and D-galactosamine (D-GalN) have been widely used to study the pathogenesis of human acute liver failure (ALF) and drug development. Our previous study reported that oyster (Crassostrea gigas) hydrolysate (OH) had a hepatoprotective effect in LPS/D-GalN-injected mice. This study was performed to identify the hepatoprotective effect of the tyrosine-alanine (YA) peptide, the main component of OH, in a LPS/D-GalN-injected ALF mice model. We analyzed the effect of YA on previously known mechanisms of hepatocellular injury in the model. LPS/D-GalN-injected mice showed inflammatory, apoptotic, ferroptotic, and pyroptotic liver injury. The pre-administration of YA (10 mg/kg or 50 mg/kg) significantly reduced the liver damage factors. The hepatoprotective effect of YA was higher in the 50 mg/kg YA pre-administered group than in the 10 mg/kg YA pre-administered group. These results showed that YA had a hepatoprotective effect by reducing inflammation, apoptosis, ferroptosis, and pyroptosis in the LPS/D-GalN-injected ALF mouse model. We suggest that YA can be used as a functional peptide for the prevention of acute liver injury.

## 1. Introduction

Acute liver failure (ALF) is the most common life-threatening disease in adults without pre-existing liver disease, and it mainly occurs in the 30s [1]. There are many causes of ALF that include hepatitis, acetaminophen overdose, toxins, autoimmune diseases, Wilson’s disease, and unknown factors. Herbal supplements cannot be free from triggers of ALF [2]. Since there are few effective treatments for ALF other than liver transplantation, studies to find strategies for the treatment and prevention of ALF using experimental animal models are continuously being performed. In the early stages of ALF, the incidence of bacterial infection is high [3,4], which might aggravate the clinical condition and prognosis [5]. An uncontrolled inflammatory response not only impairs the liver’s defenses but also causes massive cell death of hepatocytes, leading to acute liver damage and ultimately severe ALF [1,6].

A model made by the intraperitoneal injection of lipopolysaccharide (LPS) and D-galactosamine (D-GalN) has been widely used to study the pathogenesis of human ALF and drug development [7] because it shows clinically similar symptoms to ALF [8]. LPS, the major pathogenic component of Gram-negative bacteria, induces the secretion of large amounts of pro-inflammatory cytokines and ultimately causes liver injury [9,10,11]. D-GalN, a selective hepatotoxin, induces depletion of the intracellular uridine moiety, which in turn disrupts the hepatocyte RNA metabolism and results in liver injury [12,13]. D-GalN increases the sensitiveness of LPS and causes hepatotoxicity within a few hours. The LPS/D-GalN model shows typical hepatocellular death manifested by necrosis, apoptosis, autophagy, and inflammatory responses [14,15,16]. Although many studies have not been conducted, recent studies reported that cell death mechanisms by ferroptosis, pyroptosis, and necroptosis are involved in liver injury in the LPS/D-GalN model [17,18,19]. Substances that regulate signals related to the hepatocyte death mechanism are expected to be helpful in the prevention and treatment of LPS/D-GalN-induced liver injury.

Conventional drugs used to treat liver diseases, such as corticosteroids, antiviral drugs, and immunosuppressants, can cause serious adverse effects and even liver damage with long-term use [20]. A common strategy for preventing liver damage includes using substances with antioxidant and anti-inflammatory activity [21]. Natural products with antioxidant and anti-inflammatory activities, such as silymarin, were developed as hepatoprotectants [22]. However, since silymarin interacts with CYP2C9 inhibitors, caution is required when taking drugs related to CYP2C9 inhibitors [23]. It is necessary to broaden the choice of natural medicines suitable for individual patients by developing natural hepatoprotectants and therapeutic agents with fewer side effects than silymarin.

In our previous studies, oyster-derived hydrolysate (OH) showed hepatoprotective effects in a single ethanol binge model and a LPS/D-GalN-induced liver injury model [24,25]. In particular, the Tyrosine-Alanine (YA) peptide, the main component of OH, enhanced the ethanol metabolism and protected the liver from ethanol-induced toxicity [25]. YA has antioxidant and anti-inflammatory activities. Bioactive peptides affect various biological functions, and peptides have been used as therapeutic agents for various diseases for a long time [26]. Arg-Gly-Asp (RGD) peptide attenuates LPS-induced pulmonary inflammation [27] and hepatic fibrosis [28]. Currently, there are few studies on the hepatoprotective mechanism of YA against the LPS/D-GalN-induced liver injury model. Since the YA peptide is a food-derived substance, the preventive effect was first investigated before the therapeutic effect. This study was performed to determine the hepatoprotective effect of the YA peptide in the LPS/D-GalN-induced ALF model. We also compared the effects of two different concentrations of YA (10 and 50 mg/kg).

## 2. Results

### 2.1. Generation of Acute Liver Failure (ALF) Mouse Model

**Figure 1:**
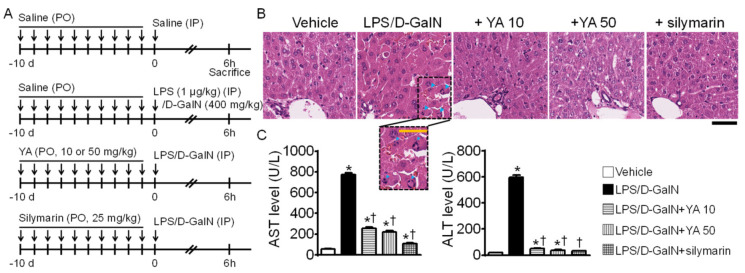
LPS/D-GalN-induced acute liver failure (ALF) mouse model. (**A**) Experimental design to determine the protective effect of YA in the ALF mouse model. Saline, YA, or silymarin was pre-administered daily for ten days by oral gavage before intraperitoneal injection of LPS/D-GalN. (**B**) LPS/D-GalN-induced pathological alterations in liver tissue attenuated by YA. The morphological changes were identified by H&E staining. The dotted rectangle representing the hemorrhage area is expanded to show. Blue arrowheads indicate nuclear fragmentation. Scale bar, 100 μm. (**C**) Effect of YA pre-administration on plasma ALT and AST levels in the LPS/D-GalN group. Data are shown as the mean ± SD (n = 10 in each group). * *p* < 0.05 compared to vehicle group. ^†^ *p* < 0.05 compared to the LPS/D-GalN group.

The method to produce an ALF mouse model and the experimental procedure to confirm the prophylactic effect of YA are summarized in Figure 1A. The ALF model was generated by the intraperitoneal injection of LPS (1 μg/kg) and d-galactosamine (400 mg/kg), and the mice were sacrificed 6 h after the LPS/D-GalN injection. The five experimental groups were divided into the vehicle, LPS/D-GalN, YA (10 or 50 mg/kg) + LPS/D-GalN, and silymarin (25 mg/kg) + LPS/D-GalN groups (each group with 10 mice). YA and silymarin were pre-administrated orally for 10 days. Saline was pre-administered instead of YA in the vehicle and LPS/D-GalN groups. Body weight was measured at the beginning and end of the experiment, and liver weight was measured immediately after sacrificing the mice. There was no significant change in body and liver weights among the experimental groups.

The morphological changes of the liver observed in the experimental groups were evaluated by hematoxylin and eosin (H&E) staining. The LPS/D-GalN group showed a remarkable increase in hemorrhage and nuclear fragmentation (dotted rectangle, Figure 1B). The morphological features of cell damage were reduced in the YA+LPS/D-GalN and silymarin+LPS/D-GalN groups. Comparing the effects of two different concentrations of YA, the cell damage was decreased more in the 50 mg/kg YA pre-administered group than in the 10 mg/kg YA pre-administered group. Silymarin (25 mg/kg), a positive control showing hepatoprotective effects, reduced the LPS/D-GalN-induced morphological features of cell damage (n = 3, Figure 1B). Alanine aminotransferase (ALT) and aspartate aminotransferase (AST) levels in the LPS/D-GalN group were significantly increased compared to the vehicle group (*p* < 0.05). In contrast, they were significantly decreased in the YA and silymarin pre-administered groups (Figure 1C, n = 10, *p* < 0.05).

### 2.2. YA Pre-Administration Attenuated Inflammatory Signals in ALF Model

**Figure 2:**
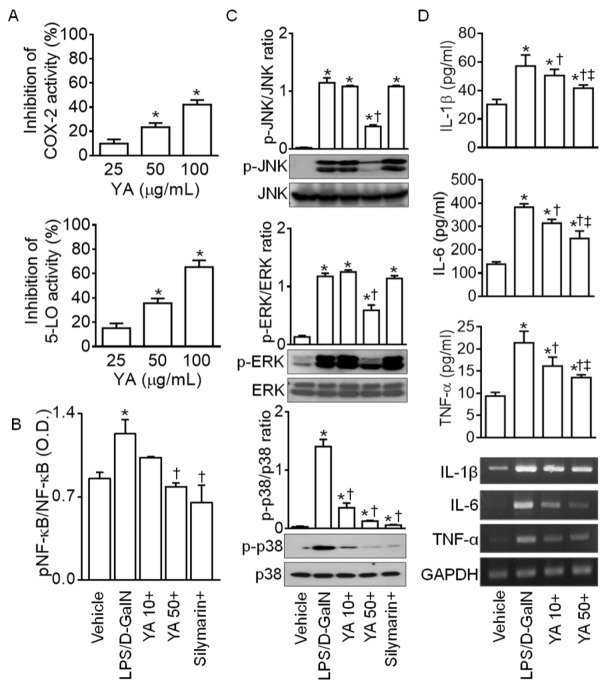
Anti-inflammatory effect of YA in LPS/D-GalN-induced ALF model. (**A**) Inhibition of cyclooxygenase-2 (COX-2) and 5-lipoxygenase (5-LO) activity by YA. * *p* < 0.05 compared to 25 µg/mL YA. (**B**) Changes in NF-κB activation. The NF-κB activity was measured using a phospho-NF-κB p65 (S536) ELISA kit. (**C**) Suppression of MAPK activation by YA. (**D**) Decrease in pro-inflammatory cytokines (IL-1β, IL-6, and TNF-α) by YA. Data are shown as the mean ± SD (n = 4 in each group). * *p* < 0.05 compared to vehicle group. ^†^ *p* < 0.05 compared to the LPS/D-GalN group. ^‡^ *p* < 0.05 compared to YA (10 mg/kg) + LPS/D-GalN group. The plus (+) sign, such as in YA10+, YA50+, and silymarin+, represents a combination of LPS/D-GalN and each substance.

YA significantly decreased the activity of the biosynthesis enzymes cyclooxygenase-2 (COX-2) and 5-lipoxygenase (5-LO), which are involved in the inflammatory process. The effect was dose-dependent (n = 4, *p* < 0.05, Figure 2A). The nuclear factor kappa-light-chain-enhancer of activated B cells (NF-κB), a key transcription factor for pro-inflammatory gene induction, was significantly activated in liver tissues obtained from the LPS/D-GalN groups compared to the vehicle group (Figure 2B, n = 4, *p* < 0.05). The NF-κB activation was significantly decreased in the 50 mg/kg YA + LPS/D-GalN and silymarin + LPS/D-GalN groups (Figure 2B, n = 4, *p* < 0.05). In the NF-κB activity, the 10 mg/kg YA + LPS/D-GalN group showed no significant difference from the vehicle and LPS/D-GalN groups.

Mitogen-activated protein kinase (MAPK) activation is related to LPS-induced inflammation [29]. Extracellular signal-regulated kinase 1/2 (ERK), c-Jun N-terminal kinases (JNK), and p38 MAPKs were significantly activated in the LPS/D-GalN group compared to the vehicle group (Figure 2B, *p* < 0.05, n = 4). ERK and JNK activation was significantly decreased in the 50 mg/kg YA + LPS/D-GalN group (*p* < 0.05), whereas p38 activation was significantly reduced in the 10 mg/kg and 50 mg/kg YA and silymarin pre-administered groups (Figure 2C, n = 4, *p* < 0.05), indicating that YA and silymarin may act through different mechanisms. Activation of NF-κB and MAPK is associated with the secretion of pro-inflammatory cytokines such as interleukin (IL)-1 β, IL-6, and tumor necrosis factor (TNF)-α [30,31]. High concentrations of IL-1β, IL-6, and TNF-α in the LPS/D-GalN group were significantly reduced in the YA + LPS/D-GalN group (Figure 2D, n = 4, *p* < 0.05). The secretion of IL-1β, IL-6, and TNF-α was more decreased in the 50 mg/kg YA pre-administered group than in the 10 mg/kg YA pre-administered group. The mRNA expression levels of IL-1β, IL-6, and TNF-α were also decreased in the YA + LPS/D-GalN groups (Figure 2D).

### 2.3. YA Pre-Administration Attenuated Apoptotic Signals in ALF Model

**Figure 3:**
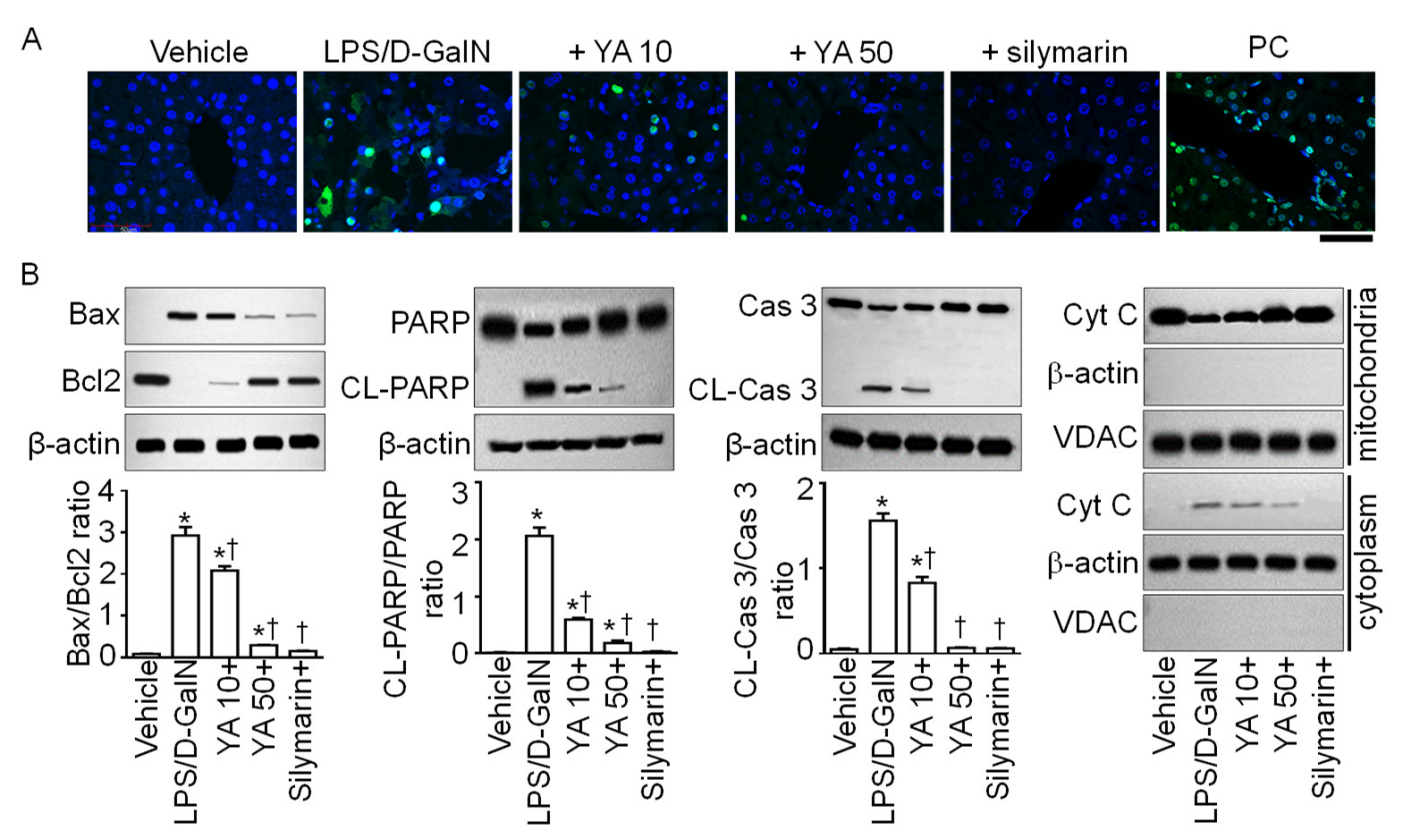
Anti-apoptotic effect of YA on liver tissues obtained from LPS/D-GalN-injected mice. (**A**) TUNEL staining. Representative fluorescence images of hepatocyte apoptosis in LPS/D-GalN group. Positive control (PC) treated with DNase I is displayed as a comparison. The cells showing green fluorescence in the nucleus are apoptotic. Scale bar, 200 μm. (**B**) Western blotting assay for detection of apoptotic signals. Pro-apoptotic Bax and anti-apoptotic Bcl2 expression levels, cleaved (CL) PARP and caspase 3 (Cas 3), and translocation of cytochrome C (Cyt C) into cytoplasm were analyzed. Data are shown as the mean ± SD (n = 4 in each group). * *p* < 0.05 compared to vehicle group. ^†^ *p* < 0.05 compared to the LPS/D-GalN group. The plus sign (+) represents a combination of LPS/D-GalN and each substance.

Apoptotic signals were analyzed in liver tissues obtained from the LPS/D-GalN-injected mice. The number of apoptotic cells exhibiting green fluorescence was increased in the LPS/D-GalN group. In contrast, the number of these cells was decreased in the YA and silymarin pre-administered groups, according to terminal deoxynucleotidyl transferase dUTP nick end labeling (TUNEL) staining, a method detecting DNA fragmentation of apoptotic cells (Figure 3A). In the LPS/D-GalN group, the B-cell lymphoma protein 2 (Bcl-2)-associated X (Bax)/Bcl2 ratio was increased; poly ADP-ribose polymerase (PARP) and caspase 3 (Cas 3) were cleaved, and mitochondrial cytochrome C was secreted into the cytoplasm. In comparison to the LPS/D-GalN group, the apoptotic signals were significantly reduced in the YA and silymarin pre-administered groups (Figure 3B, *p* < 0.05, n = 4). The inhibitory effect on LPS/D-GalN-induced apoptotic signals was higher in the 50 mg/kg YA pre-administration group than in the 10 mg/kg YA pre-administration group.

### 2.4. YA Pre-Administration Attenuated Endoplasmic Reticulum (ER) Stress and Ferroptosis and Pyroptosis Signals in ALF Model

**Figure 4:**
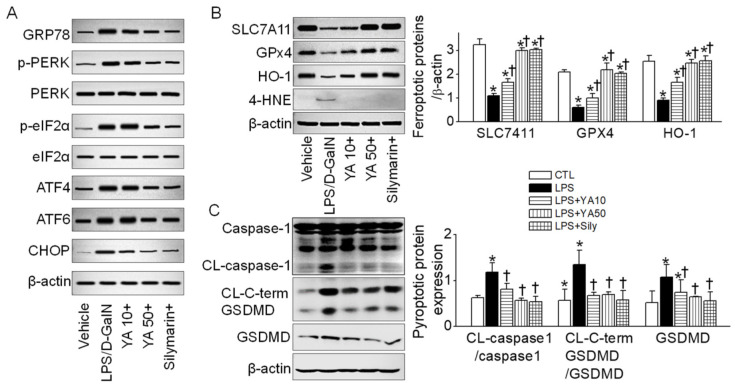
Ferroptotic and pyroptotic signals decreased by YA pre-administration. (**A**) Changes in ER stress markers. (**B**) Changes in ferroptosis markers. (**C**) Changes in pyroptosis markers. Data were shown as the mean ± SD (n = 3 in each group). (**B**,**C**) share a label representing each experimental group. * *p* < 0.05 compared to vehicle group. ^†^ *p* < 0.05 compared to the LPS/D-GalN group. The plus sign (+) means the combination of LPS/D-GalN and each substance.

ER stress is related to various cell death mechanisms. ER stress-related proteins such as GRP78, PERK, eIF2α, ATF4, ATF6, and CHOP were upregulated in the LPS/D-GalN group (Figure 4A). The upregulated ER stress markers were markedly decreased in the YA and silymarin pre-administered groups. SLC7A11, GPx4, and HO-1 suppression are linked to ferroptosis induction, while 4-HNE upregulation is related to lipid peroxidation during this process. SLC7A11, GPx4, and HO-1 protein expression levels were decreased in the LPS/D-GalN group, while the 4-HNE protein expression level was increased. The changes in the ferroptosis markers were significantly restored in the YA and silymarin pre-administered groups (Figure 4B, *p* < 0.05, n = 3).

Pyroptotic cell death was detected in the LPS/D-GalN group. The caspase-1 was cleaved, and the gasdermin D (GSDMD) was upregulated. In addition, the carboxy-terminal gasdermin-C domain cleaved in gasdermin D (CL-C-terminal GSDMD) was detected in the LPS/D-GalN group (Figure 4C). IL-1β was highly secreted in the LPS/D-GalN group (see Figure 2D). The pyroptotic signals were significantly reduced in the YA+LPS/D-GalN and silymarin+LPS/D-GalN groups (Figure 4C, *p* < 0.05, n = 3).

**Figure 5:**
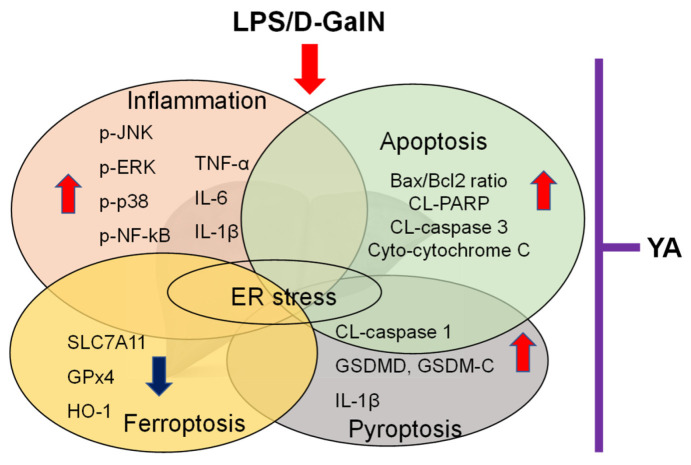
Various mechanisms were observed in liver tissues obtained from the LPS/D-GalN-induced ALF model. Pre-administration of YA reduced LPS/D-GalN-induced liver damage factors. The mechanisms are interconnected and can exacerbate liver damage.

The mechanisms involved in liver injury in LPS/D-GalN-injected mice are summarized in Figure 5.

## 3. Discussion

This study analyzed the effect of YA on previously known mechanisms of hepatocellular damage in a mouse model capable of mimicking ALF symptoms. LPS/D-GalN-injected mice used as an ALF model in this study are also referred to as models for fulminant liver failure (FLF), acute liver injury (ALI), and acute hepatitis. Previous studies reported that liver damage in LPS/D-GalN-injected mouse models is induced by multiple complex mechanisms, such as inflammation, apoptosis, necrosis, autophagy, pyroptosis, necroptosis, and ferroptosis [14,15,16,17,18,19]. However, most previous studies using LPS/D-GalN-injected mouse models analyzed one or two of the mechanisms mentioned above and reported that many hepatoprotectants proposed in those studies modulate the analyzed mechanisms. Since various factors and mechanisms cause ALF, substances that can control several mechanisms at once will be more helpful in treating ALF. Many hepatoprotective natural substances are more likely to exert their effects by regulating multiple mechanisms rather than specifically regulating a single mechanism. However, due to the lack of research, only some mechanisms of action of these substances are known. Even if the effects of these substances are excellent, it is challenging to develop new drugs or healthy functional foods if the mechanism of action is not sufficiently analyzed.

Here, we introduce a YA peptide that regulates many pathological mechanisms occurring in the LPS/D-GalN-induced ALF model. YA reduced inflammation, apoptosis, ER stress, ferroptosis, and pyroptosis in a LPS/D-GalN-injected mouse model, eventually reducing liver injury. Dipeptide YA used in this study has not been studied as much as other peptides. The mechanism of LPS/D-GalN-induced liver injury is also associated with increased autophagy [16]. However, in our study, autophagy-related signals did not show consistent results, so autophagy was excluded from the hepatoprotective mechanism of YA. Necrotic events were confirmed by H&E staining. Hepatocyte swelling along with shrinkage of the nucleus shown in the LPS/D-GalN-induced ALF model was reduced in the YA pre-administered group. Receptor-interacting protein kinase (RIPK) 1 and RIPK3, necroptosis markers, expression levels were also checked in the liver tissues obtained from the LPS/D-GalN-induced ALF model. There were no significant differences between the vehicle group and the LPS/D-GalN group. As a result, autophagy and necroptosis were excluded from YA-regulated mechanisms.

YA exerts an anti-inflammatory effect by reducing MAPK/NF-κB activity. Apoptosis plays a role in normal liver development. However, the over-activation of apoptosis may lead to hepatocellular damage [32,33]. YA exerts anti-apoptotic effects by decreasing the Bax/Bcl2 ratio, PARP and caspase 3 cleavages, and cytochrome C translocation from the mitochondria to the cytoplasm. ER stress plays a role during LPS/D-GalN-induced apoptosis in the ALF model [34,35,36]. YA decreased most of ER stress protein expression. ER stress is related to apoptosis, inflammation, and pyroptosis [37,38]. Ferroptosis agents cause ER stress responses, which play an essential role in the cross-talk between ferroptosis and other types of cell death [39]. ER stress appears to mediate many kinds of cell death. Dysregulation of ferroptosis has also been associated with various liver diseases [40]. Ferroptosis occurs mainly due to downregulated system x_c_ activity, inhibited glutathione peroxidase 4 (GPX4), and increased lipid ROS [41]. Functional subunit solute carrier family member 11 of system x_c_ (SLC7A11), GPx4, and HO-1 protein expression are reduced in the LPS/D-GalN-induced liver injury model [19,42]. YA pre-administration reversed ferroptotic signals in the LPS/D-GalN-induced ALF model. Apoptosis, necroptosis, and pyroptosis can be switched by some molecules. GSDMD is a pore-forming protein that promotes pyroptosis and the release of pro-inflammatory cytokines [43]. GSDMD-mediated hepatocyte pyroptosis extends the inflammatory response to ALF by upregulating monocyte chemotactic protein 1/CC chemokine receptor-2 to recruit macrophages [17,18]. YA pre-administration also decreased the upregulation of GSDMD, caspase 1 activation, C-terminal of GSDMD cleavage in the LPS/D-GalN induced ALF model. These signals are intricately intertwined in the ALF model and will act in complex ways. In addition, the analyzed mechanism may not be perfect. Other mechanisms will work. YA regulates various mechanisms, which can occur in the LPS/D-GalN-induced ALF model. Involvement in multiple mechanisms can be either an advantage or a disadvantage. The advantage is that it can be effective because it can block numerous pathways that can act as mechanisms of liver damage in the ALF model at once. The disadvantage is that since it blocks several pathways, the probability of side effects can be high, and YA may not work specifically for the ALF model. However, in terms of side effects, since YA is a peptide derived from natural products, it is considered that the possibility of side effects is low.

YA was used as a standard material for OH. Although several peptides have been suggested as standard materials in the OH, YA has advantages over other peptides. YA is readily available to be used because YA is synthesized and sold by several companies, including Sigma-Aldrich (#T5128). Short peptides produced from proteins that have biological activity beyond their nutritional value are known as bioactive peptides. To achieve their “bioactive” roles, these peptides must be released by proteolysis (in vivo digestion, in vitro enzymatic hydrolysis, or bacterial fermentation) [44]. YA was released from oysters by enzymatic hydrolysis. Our previous studies demonstrated that OH produced by in vitro enzymatic hydrolysis of oysters contained various bioactive peptides such as TAY, VK, KY, FYN, and YA and displayed antihypertensive, anti-inflammatory, antidiabetic, antioxidative, and hepatoprotective effects in in vitro and in vivo tests [24,25,45,46,47]. YA can be a bioactive peptide.

In the case of peptides, when administered orally, they are broken down into amino acids in the gastrointestinal tract, which may weaken their effectiveness. When comparing the effects of oral and intraperitoneal administrations in a preliminary study, the YA effect was slightly higher when injected intraperitoneally, indicating that the peptide may be digested into amino acids without being wholly absorbed when YA was administered orally. However, it is thought that this disadvantage can be overcome by intramuscular, subcutaneous, or intravenous injection. If YA is catabolized, tyrosine and alanine will be produced. Tyrosine and alanine are non-essential amino acids. Alanine is the most common amino acid catabolized by the liver in mammals, and it contributes the most to the gluconeogenesis of the 15 glucogenic amino acids [48,49]. To clear the N metabolites generated by amino acid catabolism, peripheral tissues such as skeletal muscle produce alanine and glutamine as nitrogen carriers in the blood, which are then taken up by the liver and gut and safely disposed of ureagenesis, resulting in glucose production from alanine [48,49]. Furthermore, the ALT expressed in the liver is responsible for the alanine-pyruvate interconversion [50]. When hepatocytes are damaged, ALT is released into the bloodstream, increasing serum ALT activity [51]. In an ALF rat model treated with D-GalN, alanine administration was found to lower plasma levels of ALT and total bilirubin dramatically [52]. In a CCl_4_-induced hepatocyte necrotic rat model, alanine administration was shown to reduce the ALT rise and histological liver damage [53]. In addition, alanine treatment dramatically reduced lactate dehydrogenase levels in D-GalN-treated rat hepatocytes [53]. When dietary tyrosine levels are low, the liver can produce tyrosine by hydroxylating phenylalanine. Tyrosine can become an essential amino acid in conditions where the liver fails. Tyrosine shortage can cause net protein catabolism and muscle wasting, so it is important to get enough [54]. Furthermore, tyrosine that is overused is oxidized. Tyrosine is a ketogenic and glucogenic amino acid. Both glucose and fatty acids can be produced by tyrosine [54]. Blood tyrosine levels are supposed to rise as a result of all-cause liver disease [55]. However, the exact mechanism is not known, and there is little evidence that tyrosine has a direct influence on liver disease. The YA concentration in the gastrointestinal system did not vary significantly in the simulated digestion experiment, suggesting that YA can be absorbed into the blood without significant loss [45].

In addition, when comparing the hepatoprotective effect of YA between the group pre-administered with YA once a day for 10 days (10 days YA group) and the group pre-administered with YA once a day (1 day YA group), the hepatoprotective effect of YA was slightly lower in the 1 day YA group than the 10 day YA group, with a reduction in liver damage. At 10 and 50 mg/kg concentrations of YA, both concentrations effectively reduced liver damage in the LPS/D-GalN-induced ALF model, except for effects on ERK and JNK activation. The hepatoprotective effect was higher in the 50 mg/kg YA pre-administered group than in the 10 mg/kg YA pre-administered group. In addition, significant activation of ERK and JNK in the 50 mg/kg YA pre-administered group could act as a signaling pathway distinct from the silymarin pre-administered group. A single ethanol binge model with 50 mg/kg YA demonstrated a hepatoprotective effect [25]. Therefore, we compared the effect of low-dose (10 mg/kg) and high-dose (50 mg/kg) YA in the LPS/D-GalN-induced ALF model. It can become a more effective functional food and is more likely to be used as a pharmaceutical if it has an effect at a low concentration. Although less effective than 50 mg/kg YA, 10 mg/kg YA had a hepatoprotective effect, suggesting that it could be developed as a medication. YA did not cause hepatotoxicity at 50 mg/kg, and it may affect other mechanisms that were not fully explored in this study.

Food-derived bioactive peptides and peptide-rich protein hydrolysates could provide a safe alternative to synthetic pharmaceuticals for the prevention and treatment of acute and chronic diseases with fewer side effects. The positive effect of YA was confirmed in the acute inflammation models, but its effect should also be analyzed in the chronic models. The substances that modulate multiple mechanisms may be effective because they can control complex mechanisms that can coexist in a single disease. However, it will be necessary to investigate continuously the side effects of the substance on normal tissues. Our findings suggest that YA can be a hepatoprotectant in acute liver injury, such as ALF, FLF, and acute hepatitis as a bioactive peptide.

## 4. Materials and Methods

### 4.1. Preparation of YA Peptide

Crassostrea gigas specimens (length, 5.8 ± 0.4 cm; height, 3.2 ± 0.4 cm; body weight (BW), 9.8 ± 2.1 g) were harvested from a fish farm in Tongyeong (South Korea) in 2018–2019, frozen, and preserved for 1–2 years. The preparation of the oyster hydrolysate (OH) and YA was conducted according to a previous protocol [25]. The amino acid sequence of the purified peptide fragment in OH is determined using LC/MS/MS. The sequenced peptide YA was synthesized with a purity of 95% or higher to test their function. In addition, we validated histological changes in the liver and changes in liver enzymes (alanine aminotransferase, ALT; aspartate aminotransferase, AST) using synthetic YA purchased from Sigma-Aldrich (St. Louis, MI, USA).

### 4.2. Measurement of Cyclooxygenase-2 (COX-2) and 5-Lipoxygenase (5-LO) Inhibition Activity

The percentages of COX-2 inhibition and 5-LO inhibition of YA were measured according to the previous protocols [24]. Briefly, the assay mixture for COX-2 contained 450 µL of Tris-HCl buffer (pH 8.0, 100 mM), 100 µL of hematin (150 mM), 100 µL of ethylene-diamine-tetraacetic acid (EDTA, 30 µM), 200 µL of COX-2 (40 U/mL), and 100 µL of YA. The mixture was incubated for 15 min at room temperature. The reaction was initiated by adding 20 µL of arachidonic acid (20 mM) and 25 µL of N,N,N’,N’-tetramethyl-ρ-phenylenediamine (TMPD, 10 mM) and evaluated after 5 min at 590 nm. To measure 5-LO activity, 200 µL of the enzyme solution (160 U/mL) were prepared in a 0.2 M boric acid buffer (pH 9.0), mixed with 50 µL of YA (1, 3, 5, and 100 mg/mL in boric acid buffer), and then incubated at room temperature for 3 min. The reaction was initiated by adding 250 µL of the substrate solution (100 µM of linoleic acid) and evaluated for 2 min at 234 nm using the VERSAmax microplate reader (Molecular Devices, San Jose, CA, USA).

### 4.3. LPS/D-GalN-Induced ALF Model

The animal experiments were carried out in compliance with the animal care and use committee guidelines at Gyeongsang National University (GNU-151208-M0068). Male C57BL/6 mice (7 weeks old) were purchased from Koatech Co. (Animal Breeding Center, Pyongtaek, Korea). Animals were kept on a 12 h light/dark cycle in a specific pathogen-free area with food and water freely available in the animal facility for 1 week before the experiment. All experimental animals were randomly separated into five groups as follows: Saline, LPS (1 µg/kg) + D-GalN (400 mg/kg), LPS/D-GalN + YA (10 mg/kg), LPS/D-GalN + YA (50 mg/kg), and LPS/D-GalN + silymarin (25 mg/kg). YA and silymarin were pre-administered for 10 days before LPS/D-GalN by oral gavage. LPS/D-GalN was injected intraperitoneally. Blood and tissues were collected 6 h after LPS/D-GalN injection. Liver tissues were quickly isolated and placed into a deep freezer at −80 °C or a 4% paraformaldehyde solution for further experimentation.

### 4.4. Measurement of Alanine Aminotransferase (ALT) and Aspartate Aminotransferase (AST) Levels

ALT and AST levels in the serum were measured by GC Labs (Yongin, Korea), which uses the International Federation of Clinical Chemistry standard method. ALT and AST levels were measured and analyzed according to previous methods [25].

### 4.5. Hematoxylin and Eosin (H&E) Staining

Histological changes in the liver tissue were analyzed by H&E staining (Sigma Aldrich., St Louis, MO, USA). Mice were perfused with a fixative solution containing 4% paraformaldehyde solution, and the liver was isolated and incubated in the same fixative solution overnight at 4 °C. The liver tissues were embedded in paraffin after washing three times. The paraffin blocks were sectioned to a thickness of 5 μm and air-dried on gelatin-coated slides. For H&E staining, the paraffin was removed from the liver tissue sections with xylene, and the tissue sections were rehydrated with graded alcohol series (100% to 70% EtOH). The liver tissue section was washed with tap water for 5 min, and the section slide was immersed in hematoxylin solution for 5 min. After checking the degree of hematoxylin staining, eosin staining was performed for 1 min. The sections were dehydrated through a graded series of EtOH (70% to 100% EtOH, each 3 min), removed from xylene, and mounted with mounting medium (Fisher Chemical, Geel, Belgium). The stained part was photographed using a BX61VS microscope (Olympus, Tokyo, Japan).

### 4.6. TUNEL Staining

The apoptotic signal in the testes was assessed using the DeadEnd Fluorometric TUNEL System (Promega, Madison, WI, USA) according to the manufacturer’s protocol. The TUNEL staining was carried out as described previously [56]. Deparaffinized liver tissue sections were fixed in 4% paraformaldehyde in PBS for 15 min at room temperature, washed three times in PBS, and permeabilized with 20 μg/mL proteinase K solution for 10 min at room temperature. After three washes in PBS, the slides were refixed in 4% paraformaldehyde for 5 min at room temperature. The slides were washed in PBS for 5 min and equilibrated in an equilibration buffer for 10 min. The liver tissues on the slides were labeled with a TdT reaction mix for 60 min at 37 °C in a dark, humidified chamber. The reaction was stopped with a 2 × SSC solution, followed by washing three times in PBS. Counterstaining was carried out by incubating with 5 μg/mL PI for 10 min at room temperature in the dark. TUNEL-positive cells were observed using a confocal laser scanning microscope (Olympus).

### 4.7. RT-PCR

Total RNA isolated from liver tissues was used to synthesize first-strand cDNA using a reverse transcriptase kit (DiaStartTM RT kit; SolGent, Daejeon, Korea) for RT-PCR and real-time PCR. As previously mentioned, the procedure for RT-PCR was performed [57]. Table 1 shows the primer sequences used to detect mRNA of IL-1β, IL-6, TNF-α, and glyceraldehyde-3-phosphate dehydrogenase (GAPDH). GAPDH was used as a loading control. The PCR conditions included an initial denaturation at 94 °C for 5 min, followed by 30 cycles of 94 °C for 30 s, 58 °C for 30 s, and 72 °C for 30 s, and a final extension step at 72 °C for 10 min.

### 4.8. Western Blot Analysis

A Western blot analysis of total, cytoplasmic, and mitochondrial proteins was performed as described previously [25]. The total protein was isolated from liver tissue using the RIPA buffer (25 mM Tris-HCl (pH 7.4), 150 mM NaCl, 1% NP-40, 1% deoxycholate, 0.1% sodium dodecyl sulfate (SDS); Thermo Fisher Scientific, Carlsbad, CA, USA) containing a 1× protease inhibitor cocktail (Roche Diagnostics, Indianapolis, IN, USA). According to the manufacturer’s protocol, mitochondrial and cytosolic fractions were isolated using a mitochondria isolation kit for the tissue (Thermo Fisher Scientific). Equal amounts (30 μg) of protein were analyzed among experimental groups. Equal volumes of the proteins and 2× SDS sample buffer were mixed, loaded on 10% SDS-polyacrylamide gel, and separated by electrophoresis for 120 min at 120 V. Then, the gel was transferred to a polyvinylidene difluoride membrane (Millipore, Billerica, MA, USA) for 1 h at 100 V using a wet transfer system (Bio-Rad, Hercules, CA, USA). The membranes blocked with 5% (*w*/*v*) fat-free dry milk in TBS with tween-20 at room temperature for 60 min were incubated with anti-Bax (1:200 dilution; Santa Cruz Biotechnology, Dallas, TX, USA), anti-Bcl-2 (1:200 dilution; Santa Cruz Biotechnology), anti-cytochrome C (1:1000; Cell Signaling, Danvers, MA, USA), anti-VDAC (1:1000; Cell Signaling, Danvers, MA, USA), anti-caspase-3 (1:1000; Cell Signaling), anti-GRP78 (1:1000 dilution, Abcam., Cambridge, UK), anti-pERK (1:200 dilution; Santa Cruz Biotechnology), anti-p-pERK (1:200 dilution; Santa Cruz Biotechnology), anti-elF2α (1:1000; Cell Signaling), anti-p-elF2α (1:1000; Cell Signaling), anti-ATF4 (1:200 dilution; Santa Cruz Biotechnology), anti-ATF6 (1:1000; Cell Signaling), anti-CHOP (1:200 dilution; Santa Cruz Biotechnology), anti-HO-1 (1:200 dilution; Santa Cruz Biotechnology), anti-SLC7A11/xCT (1:1000, arigo Biolaboratories Corp., Hsinchu City, Taiwan), anti-GPx4 (1:1000, arigo Biolaboratories Corp), anti-4-HNE (1:1000, arigo Biolaboratories Corp), anti-caspase 1 (1:1000, Adipogen Corporation, San Diego, CA, USA), anti-GSDMD (1:1000, Abcam), anti-cleaved C-terminal GSDMD (1:1000, Abcam), and anti-β-actin antibody (1:5000 dilution; Thermo Fisher Scientific) at 4 °C overnight. After the primary antibody incubation, it was incubated with a secondary HRP-conjugated anti-rabbit or anti-mouse antibody at 1:10,000 (Assay Designs, Ann Arbor, MI, USA). Immuno-positive bands were enhanced with chemiluminescence (EzWestLumi plus; ATTO Gentaur, Tokyo, Japan) and visualized using the iBright CL1500 imaging system (Thermo Scientific Fisher/ Life Technologies Holdings Pte Ltd., Singapore).

### 4.9. Measurement of IL-1β, IL-6, and TNF-α Concentrations in Liver Tissues

According to the manufacturer’s protocol, the concentrations of the pro-inflammatory cytokines IL-1β, IL-6, and TNF-α in the liver tissues were quantified using an ELISA kit (R&D system, Minneapolis, MN, USA). The protocol was previously described [25]. The absorbance of the plates at 450 nm was read with a microplate reader (Molecular Devices).

### 4.10. Measurement of Total and Phospho-NF-ĸB p65 Protein

The semi-quantitative measurement of NF-κB p65 (pS536) and total NF-κB p65 protein was performed using an ELISA kit (NF-κB p65 (pS536 + Total), Abcam) according to the manufacturer’s protocol. The 100 mg of liver tissues were homogenized in cold 1× Extraction Buffer PTR (Abcam). The homogenates were incubated in ice for 20 min and subjected to centrifugation at 18,000× *g* for 20 min at 4 °C (Eppendorf Centrifuge 5424R, Eppendorf AG). After centrifugation, the supernatant was transferred to a clean tube and provided as tissue lysates. The protein concentration of tissue lysate was quantified using the BCA assay kit. Then, the tissue lysates were diluted to the desired concentration using 1× Extraction Buffer PTR. The diluted tissue lysates (50 µL), antibody cocktail (the mixture of Capture Antibody NF-κB p65 (pS536) or NF-κB p65 total with Detector Antibody) were added to a 96-well plate. The plates were covered with an adhesive strip, incubated for 1 h at room temperature, and washed three times with 1× wash buffer PT. Then, 100 µL of TMB substrate were added and incubated at room temperature for 15 min in the dark on a plate shaker set to 400 rpm. The reaction was quenched by adding 100 µL stop solution, and the absorbance of the plates was read at 450 nm with a microplate reader (Molecular Devices, Sunnyvale, CA, USA).

### 4.11. Data Analysis and Statistics

The images of the Western blots and agarose gel were captured using an iBright CL1500 imaging system (Thermo Scientific Fisher/Life Technologies Holdings Pte Ltd.). The bands were quantified by ImageJ software (version 1.49, National Institutes of Health, Bethesda, MD, USA). Data are presented as the mean ± standard deviation (SD). The one-way ANOVA/Bonferroni test or the Kruskal–Wallis/Mann–Whitney test was selected after the normality test to analyze differences among groups (OriginPro2020, OriginLab Corp., Northampton, MA, USA). A *p* < 0.05 value was considered as the statistical significance criterion.

## 5. Conclusions

Our findings demonstrate that YA regulates a complex mechanism induced by LPS/D-GalN. YA has anti-inflammatory, anti-apoptotic, anti-pyroptotic, and anti-ferroptotic effects against LPS/D-GalN-induced hepatic inflammation and cell death. YA may be a potential marine anti-inflammatory and antioxidative agent for treating acute liver diseases such as ALF.

## Figures and Tables

**Table 1:** Primer sequences used for PCR.

Gene Name	GenBank Acc. No.	Primer Sequences (5′–3′)	Expected Size (bp)
*IL1* *b*	NM_008361.4	Sense: GTTGACGGACCCCAAAAGAT Antisense: TCGTTGCTTGGTTCTCCTTG	440
*IL6*	NM_031168	Sense: CTTCACAAGTCCGGAGAGGAG Antisense: TGGTCTTGGTCCTTAGCCACT	489
*T* *nf*	D84199	Sense: CAGCCTCTTCTCATTCCTGCT Antisense: TGTCCCTTGAAGAGAACCTGG	339
*GAPDH*	NM_017008.4	Sense: CTA AAG GGC ATC CTG GGC Antisense: TTA CTC CTT GGA GGC CAT	201

## Data Availability

The study did not report any data.
